# The impact of lipoprotein(a) determination in cardiovascular risk stratification among people living with HIV

**DOI:** 10.3389/fcvm.2026.1899273

**Published:** 2026-08-21

**Authors:** Carmen Ramos Alejos-Pita, Diego José Rodríguez Torres, Tatiana Mata Forte, Beatriz Tendero de la Asunción, Joaquín Enrique Gómez Sanz, Juan José Reyes Luján, Matteo Romano, Edurne López Soberón, Francisco Javier Membrillo de Novales, Miriam Estébanez Muñoz

**Affiliations:** 1 Department of Cardiology, Central Defense Hospital Gómez Ulla, Madrid, Spain; 2 University of Alcalá, Madrid, Spain; 3 CBRN and Department of Infectious Diseases, Central Defense Hospital Gómez Ulla, Madrid, Spain; 4 General Biochemistry Section, Department of Clinical Analysis, Central Defense Hospital Gómez Ulla, Madrid, Spain; 5 Research Section, Department of Education, Central Defense Hospital Gómez Ulla, Madrid, Spain; 6 Department of Radiology, Central Defense Hospital Gómez Ulla, Madrid, Spain

**Keywords:** antiretroviral therapy, cardiovascular risk, coronary computed tomography angiography, HIV infection, lipoprotein(a)

## Abstract

**Objectives:**

Human immunodeficiency virus (HIV) infection has become a chronic condition due to antiretroviral therapy (ART), which has increased life expectancy but is also associated with a higher burden of cardiovascular disease (CVD) in people living with HIV (PLHIV). Lipoprotein(a) [Lp(a)] is an independent cardiovascular risk biomarker; however, its role in PLHIV remains unclear. This study evaluated the relationship between Lp(a) and cardiovascular risk and explored its association with subclinical coronary artery disease.

**Methods:**

Cross-sectional study included PLHIV aged ≥40 years on stable ART with sustained viral suppression. Individuals with prior cardiovascular events or type 1 diabetes or long-standing type 2 diabetes (≥10 years) were excluded. Lp(a) levels and clinical data were collected. Cardiovascular risk was estimated using validated clinical scores; participants with ≥10% risk underwent coronary computed tomography angiography (CTA) to assess coronary anatomy.

**Results:**

A total of 69 patients were included (81% male; mean age 54 years). The median Lp(a) level was 23.4 nmol/L (IQR: 8.2–81.6; mean: 66.7 nmol/L); 29% had ≥75 nmol/L and 23% ≥ 105 nmol/L. A non-significant inverse association was observed between Lp(a) levels and ART duration (*r* = −0.209; *p* = 0.08). Of 46 participants with a Framingham score ≥10%, 23 underwent coronary CTA. Among those with elevated Lp(a), a higher proportion had vulnerable plaques on CTA, although this was not statistically significant; because only 5 participants with elevated Lp(a) underwent CTA, this finding should be regarded as hypothesis-generating.

**Conclusions:**

In this cohort of virologically suppressed PLHIV, a non-significant inverse trend (*p* = 0.08) was observed between ART duration and Lp(a) levels. In the subgroup of moderate-to-high cardiovascular risk, the prevalence of coronary plaque vulnerability features was higher among those with elevated Lp(a), although the small number of imaging studies means this observation should be considered hypothesis-generating rather than indicative of an established biological association.

## 1 Introduction

Human immunodeficiency virus (HIV) infection has transitioned into a chronic condition due to the widespread use of antiretroviral therapy (ART), which has significantly improved survival. As life expectancy increases, people living with HIV (PLHIV) experience a higher burden of non–AIDS-related comorbidities, particularly cardiovascular disease (CVD), which is now a leading cause of morbidity and mortality in this population (1, 2). Despite extensive research, the mechanisms underlying the elevated cardiovascular risk in PLHIV remain incompletely understood, and evidence indicates that this population is at increased risk of developing subclinical atherosclerosis, even after adjustment for traditional risk factors (3, 4). Given this elevated cardiovascular burden in PLHIV, it is essential to identify additional biomarkers that may contribute to risk stratification. Among these, lipoprotein(a) [Lp(a)] has emerged as an important and causal factor in atherosclerotic cardiovascular disease.

Lp(a) is a low-density lipoprotein (LDL)-like particle composed of apolipoprotein(a) bound to apolipoprotein B-100, with well-established proatherogenic and pro-inflammatory properties and is recognized as an independent causal risk factor for atherosclerotic cardiovascular disease (5, 6). Lp(a) concentrations are largely genetically determined, exhibit marked ethnic variability, and remain stable throughout life (7, 8). Levels ≥125 nmol/L are present in approximately 20%–25% of the general population and are associated with a two- to three-fold increased risk of myocardial infarction, stroke, and calcific aortic stenosis (9, 10). Unlike LDL cholesterol, Lp(a) is minimally influenced by lifestyle measures or standard lipid-lowering therapies, and current international guidelines recommend at least one lifetime measurement to aid cardiovascular risk stratification (9). However, despite its well-established role in the general population, important questions remain regarding the behavior, clinical relevance, and potential modulation of Lp(a) levels in PLHIV. These gaps in knowledge provide the rationale for further investigation.

In people living with HIV, persistent immune activation, chronic inflammation, and endothelial dysfunction remain present even in the context of sustained viral suppression. These factors may potentiate the proatherogenic and proinflammatory properties of Lp(a) (8), suggesting that its impact on cardiovascular risk could be amplified in this population. Long-term antiretroviral exposure, together with persistent immune activation, further contributes to the cardiovascular burden of PLHIV through metabolic and endothelial mechanisms (11). Previous studies have reported heterogeneous results regarding Lp(a) concentrations in PLHIV, with some showing higher levels than in HIV-negative individuals, while others have not observed significant differences. Recent work has also described inverse associations between Lp(a) levels and coronary endothelial function (12, 13), as well as increased perivascular inflammation on coronary imaging (14). However, despite these emerging insights, the relationship between Lp(a) and coronary plaque morphology in PLHIV has not been thoroughly investigated, and the potential influence of long-term antiretroviral therapy on Lp(a) metabolism remains insufficiently understood.

The primary objective of this study was to determine the relationship between Lp(a) levels and HIV-related clinical variables and validated cardiovascular risk scores in a contemporary cohort of PLHIV receiving stable ART. A secondary objective was to examine the association between Lp(a) levels and the presence of subclinical coronary artery disease, assessed by coronary computed tomography angiography (CTA). Coronary CTA is particularly informative in PLHIV because it detects and characterizes non-calcified and high-risk plaque, which is disproportionately prevalent in this population and is not captured by calcium scoring alone.

## 2 Materials and methods

### 2.1 Study design and population

This single-center, cross-sectional study consecutively enrolled PLHIV from the monographic consultation at Hospital Gómez Ulla in Madrid, who met all inclusion criteria and none of the exclusion criteria. Inclusion criteria were: age ≥40 years, stable antiretroviral therapy for more than 6 months, suppressed HIV viral load (<50 copies/mL), and provision of written informed consent. Exclusion criteria included a history of cerebrovascular and/or cardiovascular events, a diagnosis of type 1 diabetes mellitus (DM), or type 2 DM of more than 10 years' duration (or shorter duration in the presence of additional cardiovascular risk factors) in order to avoid including individuals with pre-existing high or very high cardiovascular risk. Recruitment began on March 22, 2022, and continued for one year. Cardiovascular risk was assessed using the Framingham Risk Score, and those with a score ≥10% were offered coronary CTA, provided there were no contraindications to the test (e.g., renal insufficiency, contrast agent allergy, or refusal to provide specific informed consent for the procedure). This study was designed as an exploratory, hypothesis-generating cross-sectional analysis whose primary aim was to estimate the prevalence and distribution of Lp(a) in virologically suppressed PLHIV; the imaging sub-study was not powered to compare plaque features across Lp(a) categories. Coronary CTA was restricted to participants at moderate-to-high estimated cardiovascular risk to avoid unnecessary radiation and contrast exposure in low-risk individuals, which explains the limited number of imaging studies available for the subgroup comparison.

### 2.2 Study variables

Demographic data, HIV-related variables, ART information, traditional cardiovascular risk factors, statin use, and lipid profile parameters were collected for all participants. Statin therapy, when present, was prescribed according to routine clinical practice and guideline-based recommendations for the management of dyslipidemia in PLHIV at the time of recruitment.

Importantly, Lp(a) levels were determined from a blood sample obtained after the participant's inclusion in the study. This approach allowed for an unbiased evaluation of Lp(a) distribution within the cohort and facilitated estimation of its prevalence among PLHIV with virological suppression. Lp(a) levels were determined using a particle-enhanced immunoturbidimetric assay (Roche Diagnostics), with results initially expressed in nmol/L according to current recommendations10 (≥75 nmol/L: moderately elevated; ≥105 nmol/L: elevated according to the 2024 ESC/EAS threshold; and ≥125 nmol/L: highly elevated).

For all patients, the following cardiovascular risk scores were calculated: Framingham Risk Score, SCORE2, REGICOR, and the Data Collection on Adverse Events of Anti-HIV Drugs (D:A:D) equation. Although SCORE2 and REGICOR are contemporary tools widely used in European populations, these models are not validated specifically in PLHIV. The D:A:D score was calculated when sufficient clinical data were available; however, it could not be applied in all participants due to missing historical variables, particularly among long-term survivors. Therefore, for consistency and comparability across the cohort, the Framingham Risk Score—which has been previously used in HIV research—was selected to define participants at moderate-to-high risk (≥10%) eligible for coronary computed tomography angiography (CTA).

### 2.3 Coronary computed tomography angiography

All patients initially underwent ECG-synchronized, non-contrast computed tomography for coronary artery calcium (CAC) scoring, using standardized acquisition parameters (detector collimation: 64 × 1.5 mm; tube voltage: 120 kV). The Agatston score was calculated to assess baseline atherosclerotic burden. A score ≥400 was used to identify individuals at high cardiovascular risk. Subsequently, contrast-enhanced CTA was performed using a 124-detector Philips scanner.

An iodinated contrast agent (400 mg I/mL) was administered at a flow rate of 5 mL/s, followed by a 40 mL saline flush. Automatic bolus tracking was employed, with a region of interest (ROI) placed in the ascending aorta and a threshold of 100 Hounsfield units (HU) used to trigger image acquisition during the arterial phase. The contrast volume ranged from 60 to 100 mL, adjusted according to body weight and scan duration. Image post-processing was performed using IntelliSpace Portal (Philips), a dedicated client-server platform. Curved multiplanar reformations (cMPR) and interactive oblique reconstructions were generated for detailed evaluation of coronary plaques and luminal morphology.

Coronary artery stenosis was qualitatively assessed according to the Coronary Artery Disease Reporting and Data System (CAD-RADS) and graded on a scale from 0 to 5: 0 (no visible stenosis), 1 (minimal, <25%), 2 (mild, 25%–49%), 3 (moderate, 50%–69%), 4 (severe, ≥70%), and 5 (total occlusion, 100%). Stenoses were evaluated segment by segment using the modified American Heart Association (AHA) 17-segment coronary artery model. Plaque morphology was classified into the following categories: calcified plaque, predominantly calcified plaque, mixed plaque, predominantly non-calcified plaque, and non-calcified plaque. Qualitative assessment of high-risk plaque features included evaluation for low-attenuation plaque, Napkin-Ring Sign (NRS), punctate calcification and the remodeling index (RI). Low-attenuation plaque was defined as non-calcified plaque with attenuation <130 Hounsfield units (HU). The NRS was identified as a hypoattenuating central area surrounded by a thin high-attenuation rim, indicative of positive remodeling and the presence of a necrotic core. Punctate calcifications, defined as calcifications <3 mm in diameter within the plaque, suggest microcalcifications associated with plaque instability. A RI > 1.1 was considered indicative of positive vascular remodeling and was calculated as the ratio of the maximum transverse luminal diameter at the lesion site to the diameter of the nearest reference segment (proximal or distal).

A coronary lesion was classified as a vulnerable plaque when it exhibited at least two of the following features: a lipid-rich core (low-attenuation plaque), positive remodeling, and spotty calcification. This definition aligns with the criteria for vulnerable plaque proposed by other authors (15).

Coronary CTA images were jointly reviewed by an experienced radiologist and a cardiologist with expertise in cardiovascular imaging, eliminating inter-observer variability. Both reviewers were unaware of the participants' Lp(a) levels at the time of image interpretation to avoid potential bias in plaque characterization. Standardized methodologies ensured accurate characterization of stenosis severity and reliable identification of high-risk plaque features.

### 2.4 Statistical analysis

Analyses were conducted using the R statistical software, version 4.4.1 (R Core Team, 2024).

Continuous variables were tested for normality using the Shapiro–Wilk test and expressed as median values and interquartile ranges (IQR), given the non-parametric distribution of Lp(a) and other variables. Categorical variables were described using relative frequencies (percentages). Differences in Lp(a) levels were analyzed using the Wilcoxon rank-sum test for binary variables (e.g., sex, statin use) and the Kruskal–Wallis test for comparisons across multiple groups (e.g., race, ART regimen). Spearman correlation coefficients were calculated to assess associations between Lp(a) and clinical or cardiovascular risk variables (Framingham, SCORE2, REGICOR, and D:A:D risk scores). A two-sided *p*-value < 0.05 was considered statistically significant.

In addition, an exploratory analysis was performed to compare the ability of different cardiovascular risk scores (Framingham, SCORE2, REGICOR, and D:A:D when available) to identify participants with significant coronary artery disease, defined as a coronary artery calcium (CAC) score >100, using area-under-the-curve (AUC) estimates.

### 2.5 Ethical and legal aspects

All participants provided written informed consent prior to enrollment in the study, and those undergoing CTA signed an additional procedure-specific consent. Approved by the Clinical Research Ethics Committee of Hospital Central de la Defensa Gómez Ulla (approval number 8/22) before recruitment commenced. Furthermore, the study adhered to Good Clinical Practice guidelines and the Declaration of Helsinki principles, ensured participant anonymity, and the data collected were processed in accordance with current legislation on data protection (Organic Law 3/2018 of December 5 and EU Regulation 679/2016).

## 3 Results

The study included 69 participants with no known history of cardiovascular disease. The mean age was 54 years; 81% were male, and the predominant ethnicity was Caucasian (50.7%), followed by Hispanic (39.1%). Dyslipidemia was present in 49% of participants, 33% were active smokers, 30% had hypertension and 13% had type 2 diabetes. Only two participants reported a family history of premature cardiovascular disease (Table 1).

**Table 1:** Main baseline characteristics of the study.

Variable	Total patients (*n* = 69)
Age in years, mean (SD)	54.5 (9.7)
Male, *n* (%)	56 (81.2)
Race, *n* (%)	
Caucasian	35 (50.7)
Hispanic	27 (39.1)
Black	6 (8.7)
Asian	1 (1.4)
Clinical variables:	
Family history of CVD, *n* (%)	2 (2.9)
BMI^a^, mean (SD)	26.2 (5)
Diabetes mellitus, *n* (%)	9 (13.0)
Hypertension, *n* (%)	21 (30.4)
Dyslipidemia prior to baseline visit, *n* (%)	34 (49.3)
Dyslipidemia at baseline visit, *n* (%)	54 (78.3)
Use of statins, *n* (%)	30 (43.5)
Active smoker, *n* (%)	23 (33.3)
Analytical variables:	
CD4+ T cell count cells/mcl, mean (SD)	620 (294)
CD4+ T cell nadir cells/mcl, mean (SD)	338 (231)
CD4/CD8T-cell, mean (SD)	0.9 (0.6)
LDL mg/dL, mean (SD)	115 (31)
HDL mg/dL, mean (SD)	55 (17)
CrCl mL/min, mean (SD)	86 (18)
Lp(a) nmol/L, mean (SD)	66.7 (94.5)
>75 nmol/L, *n* (%)	20 (29.0)
>105 nmol/L, *n* (%)	16 (23.2)
>125 nmol/L, *n* (%)	12 (17.4)
ApoB mg/dL, mean (SD)	94 (23)
Years of HIV infection, mean (SD)	14.2 (9.5)
Years on antiretroviral therapy, mean (SD)	11.3 (7.5)
Stage C at diagnosis, *n* (%)	10 (14.5)
ART:	
PI at baseline visit, *n* (%)	12 (17.3)
PI at some point (including in baseline visit), *n* (%)	24 (34.8)
Time in months with PI^b^, mean (SD)	37 (41)
INSTI at baseline visit, *n* (%)	48 (69.5)
RTI at baseline visit, *n* (%)	8 (11.6)
Other at baseline visit, *n* (%)	1 (1.4)

SD, standard deviation; CVD, cardiovascular disease; BMI, body mass index; cls/mcl, cells per microliter; LDL, low-density lipoprotein; mg/dL, milligrams per deciliter; HDL, high-density lipoprotein; CrCl, creatinine clearance; mL/min, milliliters per minute; Lp(a) lipoprotein(a); nmol/L, nanomole per liter; ApoB, apoprotein B; HIV, human immunodeficiency virus; ART, antiretroviral therapy; PI, protease inhibitor; INSTI, integrase strand transfer inhibitors; RTI, reverse transcriptase inhibitor.

a Value collected in 54 patients of those with lipoprotein. ^b^Value collected in 62 patients of those with lipoprotein.

The median duration of ART was 11.5 years (IQR: 8–14). The mean current CD4+ T-cell count was 642 cells/μL, and the mean CD4 nadir was 338 cells/μL. Most participants (69.5%) were receiving an ART regimen containing an integrase strand transfer inhibitor (INSTI). A total of 35% had previously received a protease inhibitor (PI), and 17.4% were receiving a PI at baseline. Additionally, 43% of participants were on statin therapy.

The median Lp(a) level was 23.4 nmol/L (IQR: 8.24–81.6), and the mean level was 66.7 nmol/L. Elevated Lp(a) levels ≥75 nmol/L were present in 29% of participants, and 23% had levels ≥105 nmol/L (the 2024 ESC/EAS risk threshold). Among the two patients with a family history of premature cardiovascular disease, Lp(a) levels were 227 nmol/L and 28 nmol/L, respectively.

No significant differences in Lp(a) levels were observed across demographic, clinical, or HIV-related variables, including statin use. Weak inverse association that did not reach statistical significance was found between Lp(a) levels and ART duration (*r* = −0.209; *p* = 0.08). Similarly, no significant associations were identified between Lp(a) and cardiovascular risk estimated by validated scores, nor with current CD4 count or CD4 nadir (Table 2), indicating that Lp(a) levels were not correlated with immunological status in this cohort.

**Table 2:** Stratified comparison of Lp(a) levels by demographic, clinical, and HIV-related variables.

Variable	Association measure	*p*-value
Age	−0.12	0.33
Sex	—	0.45
Race	—	0.35
LDL	0.15	0.22
CD4+ T cell nadir	−0.07	0.63
CD4+ T cell count	0.02	0.85
Duration since HIV diagnosis	0.05	0.66
Type of ART	—	0.59
Time with ART	−0.25	0.08
Framingham risk score	−0.13	0.30
REGICOR risk score	−0.13	0.31
SCORE2	−0.09	0.48
D:A:D risk score	−0.11	0.52

Lp(a), lipoprotein(a); LDL, low-density lipoprotein; HIV, human immunodeficiency virus; ART, antiretroviral therapy. Continuous variables were analysed using Spearman correlation coefficients. Sex was compared using the Wilcoxon rank-sum test, and race and ART regimen using the Kruskal–Wallis test.

As an exploratory analysis, we also assessed the ability of the available cardiovascular risk scores to predict the presence of coronary artery calcium (CAC > 100). Although none of the scores showed a statistically significant correlation with CAC burden, the Framingham Risk Score demonstrated the highest discriminative capacity, with an area under the curve (AUC) of 0.76 (95% CI: 0.49–1.00). The very wide confidence interval, which crosses 0.50, reflects the small number of imaging studies and precludes any firm inference regarding the comparative discriminative performance of these scores; this result should be interpreted as exploratory only.

**Figure 1:**
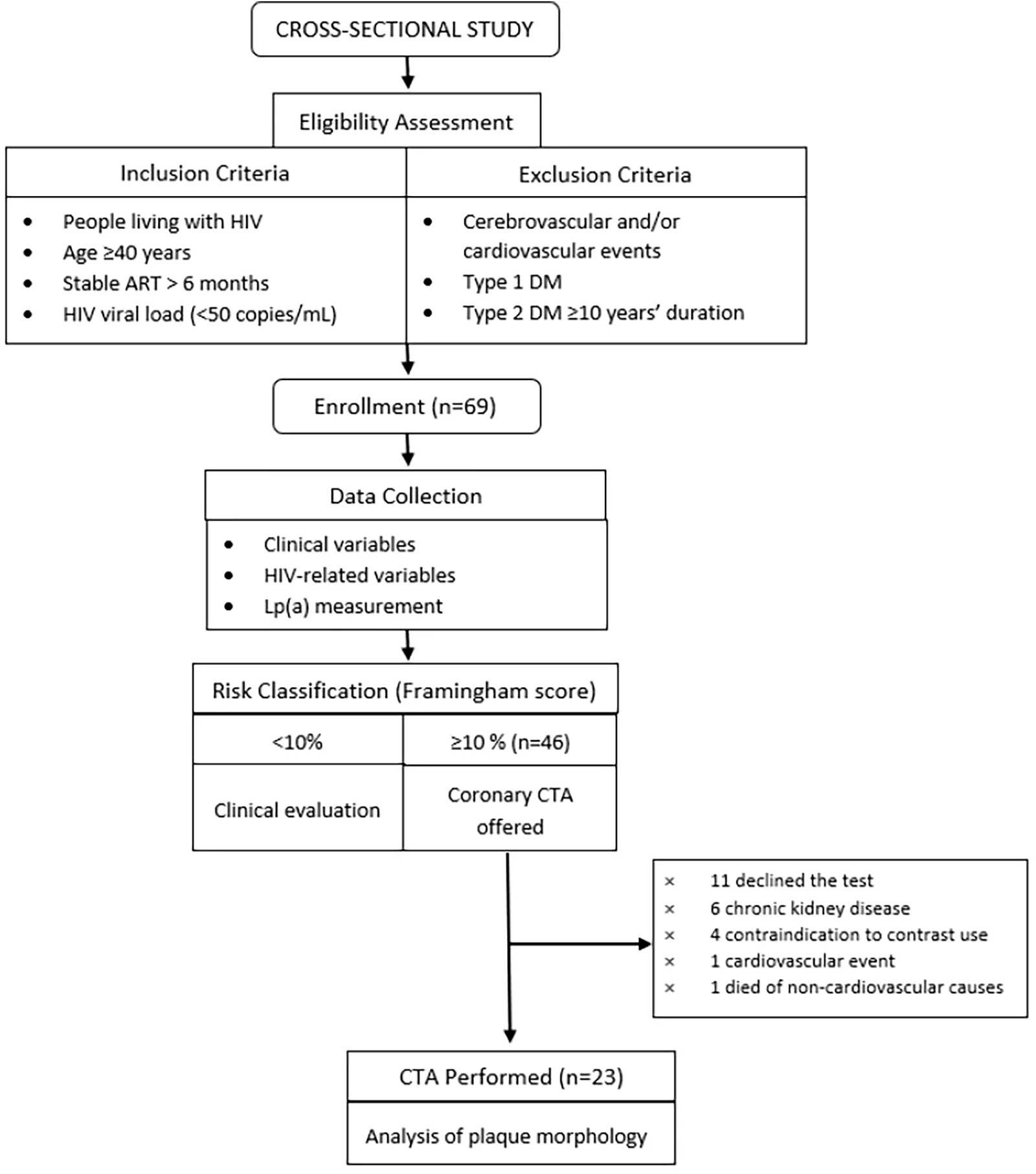
Study design and participant flow. HIV, human immunodeficiency virus; ART, antiretroviral therapy; DM, diabetes mellitus; Lp(a), lipoprotein(a); CTA, computed tomography angiography.

Of the 46 patients with a Framingham score ≥10%, 23 underwent coronary CTA. During the enrollment period, one participant classified at high cardiovascular risk experienced a cardiovascular event before undergoing CTA and was therefore not included in the imaging analysis. In addition, one participant died from a non-cardiovascular cause during follow-up and could not complete the imaging protocol. Figure 1 illustrates the reasons for patients who did not undergo the procedure. Among these participants, 52.2% had evidence of coronary stenosis. Table 3 shows the distribution of coronary lesions by arterial segment, along with their corresponding degrees of stenosis. The proximal left anterior descending artery (LAD) was the most frequently affected segment (*n* = 8), followed by the mid-LAD and proximal circumflex (Cx) segments (*n* = 5 each). Mild stenosis (<25%) was predominant. One participant showed complete (100%) occlusion of the mid-LAD, which was successfully revascularized.

**Table 3:** Coronary lesions by segment and degree of stenosis.

Segment	No. of lesions	0%–25%	50%–75%	100%
Proximal LAD	8	7	1	0
Mid LAD	5	4	0	1
Proximal LCx	5	5	0	0
Proximal RCA	4	4	0	0
Distal LCx	2	2	0	0
Proximal D1	2	0	2	0
Mid RCA	2	2	0	0
Distal LAD	1	1	0	0
RI	1	1	0	0
Mid LCx	1	1	0	0
Proximal M1	1	1	0	0
Distal RCA	1	1	0	0
Distal LCx	0	0	0	0

LAD, left anterior descending; LCx, left circumflex artery; RCA, right coronary artery; D1, first diagonal branch; RI, ramus intermedius; M1, first marginal artery.

Among participants with Lp(a) ≥ 125 nmol/L, 4 underwent CTA; among those with Lp(a) 75–124 nmol/L, 1 underwent CTA. Of these 5 individuals, 4 (80%) exhibited at least two high-risk plaque features. In contrast, among participants with Lp(a) < 75 nmol/L, 6 of 16 (37.5%) demonstrated plaque vulnerability markers. Although this difference did not reach statistical significance (Fisher's exact test *p* = 0.149), these numerical differences should be interpreted with caution given the very small number of participants in each subgroup and should be considered preliminary and hypothesis-generating. No correlation was found between Lp(a) levels and coronary artery calcium score (*r* = −0.03; *p* = 0.90).

## 4 Discussion

In this cross-sectional study of virologically suppressed PLHIV with no history of cardiovascular disease, we observed a non-significant inverse trend between Lp(a) levels and the duration of ART, a finding that should be interpreted cautiously given the exploratory nature of the analysis. No significant correlations were found between Lp(a) and current CD4+ T-cell count or CD4 nadir. However, given the limited sample size, residual confounding by immune status cannot be excluded, as a covariate may contribute to bias even in the absence of a statistically significant association. Furthermore, Lp(a) levels did not differ according to statin use, consistent with previous evidence indicating that statins have minimal or no effect on Lp(a) concentrations. Because the present study did not analyse individual ART regimens, any regimen-specific effect on Lp(a) remains speculative and would require dedicated, adequately powered analyses; the observed trend should therefore be regarded as hypothesis-generating. Nonetheless, because this correlation did not reach statistical significance, alternative explanations cannot be excluded, such as survivor bias, cohort characteristics associated with longer ART exposure, or other unmeasured confounding factors. Finally, the possibility that this inverse trend reflects a chance finding should also be considered, and confirmation in larger, adequately powered studies will be needed to determine whether this pattern represents a true biological effect or statistical variability.

In our exploratory comparison of cardiovascular risk scores, the Framingham Risk Score demonstrated a slightly better discriminative capacity for identifying individuals with significant coronary calcium burden compared with other commonly used tools. These findings, which must be interpreted with caution given the small sample size and the limited availability of complete D:A:D data, suggest that the use of Framingham to select candidates for CTA may not have introduced substantial bias in patient selection. Nonetheless, the potential for selection bias remains, particularly because individuals with longer HIV duration or incomplete historical clinical records were less likely to have calculable D:A:D scores. Larger studies incorporating complete risk-score datasets will be essential to determine the optimal risk stratification approach for identifying PLHIV who may benefit from coronary imaging.

Previous studies evaluating the relationship between HIV infection, antiretroviral therapy, and Lp(a) levels have reported heterogeneous findings. In a study conducted in the pre-ART era, Enkhmaa et al. (13) observed that HIV-infected African-American women not receiving treatment had lower Lp(a) concentrations compared with HIV-negative controls, whereas levels increased following ART initiation. In contrast, Kikuchi et al. (12) reported higher Lp(a) levels in virologically suppressed PLHIV than in HIV-negative individuals, suggesting that chronic treated HIV infection may be associated with elevated Lp(a). The mean Lp(a) level in our cohort (approximately 67 nmol/L) was slightly lower than that reported by Kikuchi et al., likely reflecting the lower proportion of individuals of African descent in our cohort. Furthermore, findings from the SPIRAL clinical trial (16) demonstrated that switching from protease inhibitor–based regimens to raltegravir resulted in a reduction in Lp(a), supporting the hypothesis that ART regimen composition, rather than HIV infection itself, may influence Lp(a) metabolism.

The biological plausibility of our findings is supported by the known proatherogenic and proinflammatory properties of Lp(a), which may be amplified in the setting of chronic HIV infection. Persistent immune activation, even in virologically suppressed individuals, contributes to endothelial dysfunction, oxidative stress, and increased expression of cellular adhesion molecules, all of which facilitate the arterial wall deposition of Lp(a). Furthermore, Lp(a) carries oxidized phospholipids and apolipoprotein(a), which stimulate proinflammatory pathways and impair fibrinolysis (10), thereby promoting the development of high-risk, unstable plaques. These mechanisms could plausibly contribute to accelerated atherosclerosis in PLHIV and could help explain the trend we observed toward a greater prevalence of vulnerable plaque features in individuals with elevated Lp(a). Collectively, these findings highlight the importance of evaluating the association between Lp(a) concentrations and coronary plaque morphology in this population.

In the subgroup of participants at moderate-to-high estimated cardiovascular risk who underwent coronary CTA, we observed a numerically higher proportion of vulnerable plaque features among participants with elevated Lp(a), although this difference did not reach statistical significance and should be viewed as exploratory. This observed pattern is consistent with findings from non-HIV populations. In the PROMISE trial (17), elevated Lp(a) levels ≥125 nmol/L were associated with both obstructive coronary artery disease and high-risk plaque morphology in asymptomatic individuals. Similarly, Dai et al. (18) demonstrated that patients with Lp(a) concentrations >125 nmol/L and multiple plaque vulnerability features had a markedly increased risk of major adverse cardiovascular events. Our observations extend these findings to PLHIV, a population in whom chronic inflammation and immune dysregulation may further potentiate Lp(a)-mediated plaque instability.

Beyond its role as a causal and genetically determined lipid biomarker, Lp(a) is increasingly recognized as a therapeutic target, with several RNA-based agents currently in advanced clinical development demonstrating reductions of more than 80% in plasma Lp(a) concentrations (19, 20). Given the elevated prevalence of Lp(a) observed in our cohort and the higher proportion of vulnerable plaque features among individuals with elevated levels, our findings underscore the potential relevance of incorporating Lp(a) into cardiovascular risk stratification algorithms for PLHIV. It should be emphasized that, in the present cohort, Lp(a) did not correlate with any of the calculated risk scores (Framingham, SCORE2, REGICOR, D:A:D); this apparent discrepancy suggests that the potential value of Lp(a) would lie in capturing residual risk not reflected by conventional equations, rather than in improving the performance of those scores, a hypothesis that requires confirmation in adequately powered studies. This approach may be particularly important in the era of emerging therapies specifically designed to lower Lp(a), which could offer novel preventive strategies for high-risk patients with HIV infection.

This study has several limitations. It was conducted in a single center with a relatively small sample size, which may limit the generalizability of the findings. In particular, the number of participants with elevated Lp(a) who underwent coronary CTA was very small, which substantially limits the statistical power of the subgroup analysis. As a result, the observed differences in plaque vulnerability features should be considered preliminary and hypothesis-generating, and interpreted with caution. In addition, the absence of an HIV-negative control group limits our ability to determine whether the associations observed between Lp(a) and subclinical coronary atherosclerosis are specific to HIV-related mechanisms or simply mirror the well-established proatherogenic effects of Lp(a) in the general population. This limitation significantly restricts the interpretability of between-group comparisons and should be addressed in future research. Future multicenter studies with larger and more diverse populations are needed to validate these findings and to determine the clinical utility of incorporating Lp(a) into cardiovascular risk stratification algorithms for PLHIV, particularly in light of emerging therapies specifically designed to lower Lp(a). Additionally, future studies integrating apolipoprotein B measurements may help further refine residual atherogenic risk in this population.

## 5 Conclusions

In conclusion, this cross-sectional study of virologically suppressed individuals living with HIV observed a weak inverse association between Lp(a) concentrations and the duration of antiretroviral therapy, suggesting a potential modulatory effect of long-term treatment exposure on Lp(a) metabolism, although this did not reach statistical significance and should be interpreted cautiously. Furthermore, in the subgroup of participants at moderate-to-high cardiovascular risk, the prevalence of coronary plaque vulnerability features was higher among those with elevated Lp(a) levels (≥75 nmol/L), highlighting a possible link between Lp(a) and subclinical atherosclerosis in this population.

Taken together, these findings support the relevance of Lp(a) as a potential biomarker for cardiovascular risk stratification in people living with HIV. Given the proatherogenic and proinflammatory properties of Lp(a), its measurement may contribute to the identification of individuals at increased risk despite viral suppression. Future prospective studies are needed to confirm these observations, elucidate underlying mechanisms, and determine whether targeted Lp(a)-lowering therapies could improve cardiovascular outcomes in this high-risk population.

## Data Availability

The raw data supporting the conclusions of this article will be made available by the authors, without undue reservation.
